# Comparing gene expression data from formalin-fixed, paraffin embedded tissues and qPCR with that from snap-frozen tissue and microarrays for modeling outcomes of patients with ovarian carcinoma

**DOI:** 10.1186/s12907-015-0017-1

**Published:** 2015-09-24

**Authors:** William H. Bradley, Kevin Eng, Min Le, A. Craig Mackinnon, Christina Kendziorski, Janet S. Rader

**Affiliations:** Department of Obstetrics and Gynecology, Medical College of Wisconsin, 8701 Watertown Plank Road, Milwaukee, WI 53226 USA; Department of Biostatistics and Medical Informatics, University of Wisconsin-Madison, Madison, WI 53792 USA; Department of Pathology, Medical College of Wisconsin, Milwaukee, WI 53226 USA; Current Address: Department of Biostatistics and Bioinformatics, Roswell Park Cancer Institute, Buffalo, NY USA

## Abstract

**Background:**

Previously, we have used clinical and gene expression data from The Cancer Genome Atlas (TCGA) to model a pathway-based index predicting outcomes in ovarian carcinoma. This data were obtained from snap-frozen tissue measured with the Affymetrix U133 platform. In the current study, we correlate the data used to model with data derived from TaqMan qPCR both snap frozen and paraffin embedded (FFPE) samples.

**Methods:**

To compare the effect of preservation methods on gene expression measured by qPCR, we assessed 18 patient and tumor sample matched snap-frozen and FFPE ovarian carcinoma samples. To compare gene measurement technologies, we correlated qPCR data from 10 patients with tumor sample matched snap-frozen ovarian carcinoma samples with the microarray data from TCGA. We normalized results to the average expression of three housekeeping genes. We scaled and centered the data for comparison to the Affymetrix output.

**Results:**

For the 18 specimens, gene expression data obtained from snap-frozen tissue correlated highly with that from FFPE samples in our TaqMan assay (r > 0.82). For the 10 duplicate TCGA specimens, the reported microarray data correlated well (r = 0.6) with our qPCR data, and ranges of expression along pathways were similar.

**Conclusions:**

Gene expression data obtained by qPCR from FFPE serous ovarian carcinoma samples can be used to assess in the pathway-based predictive model. The normalization procedures described control variations in expression, and the range calculated along a specific pathway can be interpreted for a patient’s risk profile.

**Electronic supplementary material:**

The online version of this article (doi:10.1186/s12907-015-0017-1) contains supplementary material, which is available to authorized users.

## Background

Using gene expression and clinical data from The Cancer Genome Atlas (TCGA), we previously developed a model that predicts variations in response of high-grade serous ovarian cancer to cytotoxic chemotherapies. In that publication [1], we described a method for reducing the list of genes needed to predict clinical outcomes to fewer than 100. We selected those genes from more than 10,000 possibilities by identifying genes within a core group of 12 cancer pathways [2, 3] whose variation in expression had the greatest effect on disease progression. Predictions of response to specific chemotherapeutic agents were suggested by the cumulative levels of gene expression among the 91 genes selected from the 12 pathways. Three of the pathways did not have genes identified, leaving 9 core pathways informative. We defined the predictions made by gene expression within these pathways as the Patient-Specific Risk Profile (PSRP).

Gene expression levels reported by Affymetrix microarrays and qPCR may differ significantly, creating potential difficulties for models developed on one platform and utilized in the other [4]. For example, measurements of reference RNAs from commercial sets of ~1000 genes from human brain, liver, and lung showed correlations (r) ranging from 0.45 to 0.75 when TaqMan measurements were compared with Applied Biosystems and Agilent microarray technologies [5]. The MicroArray Quality Control (MAQC) project showed correlations of r = ≥0.9 between Affymetrix and TaqMan data [6], but those measurements and correlations were made with reference RNA from a large gene set, undirected by model building or clinical practice. Moreover, the technology used to measure expression may have a greater or lesser influence on output, depending on how the genes of interest are selected. A predictive model that features only highly differentially expressed genes may more easily translate from microarray to qPCR than a model not based on large changes in expression [7]. A requirement of highly differentiated genes may not represent the biology of the disease, and the modeling we have done includes lower expressed genes in the set.

TCGA derives gene expression by placing RNA from snap-frozen tissue on an Affymetrix U133 Array platform [8]. To migrate to a more clinically functional platform, we evaluated the reliability of gene expression inputs derived by using qPCR to analyze formalin-fixed, paraffin-embedded tumor samples. Because FFPE blocks are readily available from primary debulking surgery, whereas snap-frozen tissue is not, this modified approach increases the number of patients who can potentially benefit from this profiling technique.

Two factors could significantly affect the clinical utility of our profiles when FFPE tissue samples are used: (i) differences in tissue preservation techniques altering the RNA quality and expression detection between snap-frozen and FFPE and (ii) gene expression levels differing due to alterations in technology between Affymetrix Microarray and TaqMan qPCR. Although either or both changes could have a major effect on predictive capability, the effect might be modified depending on the number of genes measured or the ways the data are analyzed for prediction. For example, the impact of variance in expression measurements could be ameliorated by aggregating multiple data points when using multiple genes for prediction [9].

In this study, we compared gene expression measurements for individual genes selected by our PSRP 91 gene assay (gene by gene) and for those same genes aggregated into the pathways of our model (pathway by pathway). We derived correlations between snap-frozen and FFPE tissue preparations and between Affymetrix U133 Microarrays and TaqMan qPCR. In addition, we used techniques for normalizing qPCR gene expression that allows us to directly compare qPCR assays with the TCGA microarray data. The result is that our PSRP 91 model developed from snap-frozen tissue on an Affymetrix platform can be tested using a qPCR outputs and FFPE specimens.

## Methods

### Study subjects

To compare tissue preservation methods, we measured gene expression from 18 patients who had both snap-frozen ovarian carcinoma samples in the Medical College of Wisconsin (MCW) gynecologic tissue bank and a case-matched FFPE sample archived by the Department of Pathology. All samples were taken during debulking surgery of patients diagnosed with Stage IIIC or IV, grade 3 serous ovarian carcinoma. Tissue samples were taken to pathology immediately after extirpation. Once assessed by a pathologist, the portion acquired for tissue banking was excised, and snap frozen in the pathology lab. The remainder was fixed in formalin and processed per standard pathology protocol. Prior to analysis, the tissues had been stored as FFPE blocks or snap frozen sections for up to 3 years. The pathology for each MCW patient was reviewed with hematoxylin and eosin (H&E) to confirm both the diagnosis and a tumor content of at least 75 % [8]. Approval from the Institutional Review Board (IRB) of the Human Research Protection Office and Medical College of Wisconsin was obtained, and all patients signed an informed consent for tissue banking.

To compare two methods of assaying gene expression, we used TCGA identification numbers to identify 10 snap-frozen tumor samples submitted to the TCGA from the tissue bank at Washington University in St. Louis. Approval from the institutional Human Research Protection Office was obtained. For each patient’s sample, TCGA had reported gene expression and annotated pathology. All of these samples were from patients with Stage IIIC, grade 3 serous ovarian carcinoma, and microarray analysis had been performed by TCGA. A qPCR expression level of the 91 genes was measured in the 10 snap frozen samples.

### Gene list

The genes whose expression we assayed were selected from a gene set constituting the 9 core pathways described previously [1]. 91 genes were chosen from our previously published PSRP results. Analysis was performed according to the 9 core pathways as well as a revised six-gene set representing the neurotrophin pathway, making a total of 10 pathways available for analysis (Additional file 1: Table S1). The subsets of genes used to define a pathway’s expression are listed in the Supplement as well (Additional file 1: Table S2). We used the housekeeping genes glyceraldehyde 3-phosphate dehydrogenase (GAPDH), hypoxanthine phosphoribosyltransferase 1 (HPRT1), and beta-D-glucuronidase (GUSB) to normalize gene expression.

### RNA isolation, cDNA, and qPCR

RNA from the snap-frozen tissue samples was extracted using an RNAqueous Kit from Life Technologies (Carlsbad, CA, USA). RNA from FFPE blocks was extracted using a RecoverAll™ Total Nucleic Acid Isolation Kit, also from Life Technologies. All RNAs were treated with Ambion TURBO DNase. RNA concentrations were determined with a Qubit™ RNA Assay Kit (Carlsbad, CA, USA), and integrity was checked on an Experion Automated Electrophoresis Station (Hercules, CA, USA).

All reagents used in cDNA synthesis and qPCR were obtained from Applied Biosystems (Carlsbad, CA, USA). RNA concentration was adjusted to 40 ng/μl for the reverse transcription reaction. cDNA synthesis was carried out with a High-Capacity cDNA Reverse Transcription Kit. About 200 ng of RNA was used in a final 10-μl RT reaction. The TaqMan® Array Plates 96 Plus were custom-made to include TaqMan Gene Expression Assays of 91 target genes and the three endogenous control genes. Best Coverage probes from Applied Biosystems were used to target the genes of interest. The assays of 91 target genes and 3 housekeeping genes were pooled at 0.2X concentration for the PreAmp reaction. cDNAs (2 μl) were preamplified in a 20-μl reaction for 10 cycles, using TaqMan PreAmp Master Mix and pooled assays. The reaction products were diluted 5-fold with 1X TE and mixed with 500 μl of TaqMan Fast Advanced Master Mix. Water was added to give a final mixture volume of 1 ml. A 10 μl aliquot of the assay mixture was added to each well of the TaqMan Gene Expression Assay Plate, and amplification was carried out on a 7500 Fast Real Time PCR System. Genes that were not detected within 34 cycles (Ct > 34) were considered unexpressed for the purpose of our evaluation.

### Normalization of affymetrix and TaqMan data

TCGA derives gene expression by extracting RNA from snap-frozen tissue and aggregating data from three different array platforms (Affymetrix U133a 2.0, Digital Gene Expression from Illumina, and a custom high-density Agilent array). We evaluated the Affymetrix results only as these were the most complete reported and had fully accessible probe information.

To compare gene expression across the two technologies (Affymetrix Microarray and TaqMan qPCR) we used the following normalization techniques for data output. For the Affymetrix data, we took the Robust Multi-array Average (RMA; Affymetrix) for each gene, and then normalized to the average of the endogenous housekeeping genes GAPDH, HPRT1 and GUSB.

**Fig. 1:**
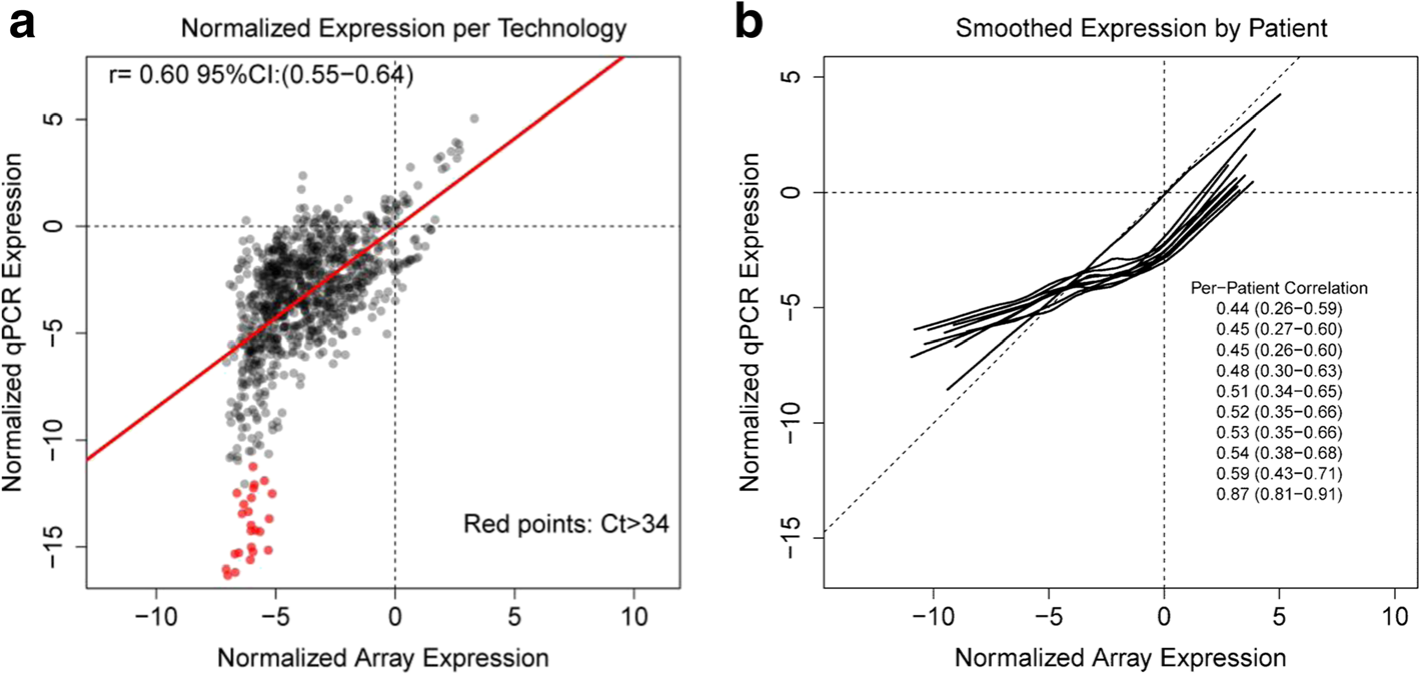
Correlation of gene expression of 91 genes from 10 snap-frozen TCGA samples measured with Affymetrix U133 microarray (X-axis) and, in the current study, with TaqMan qPCR (Y-axis). The 91 probes from the 10 samples were each normalized to the average of three housekeeping genes (GUSB, GAPDH, and HPRT1). **a** The scatterplot shows that gene-to-gene expression has similar ranges across both technologies when normalized to the same three-gene average (r = 0.60). **b** Lowess smoothing curves. Red dots signify Ct values >34 which are not included in final index measurements

For TaqMan qPCR, we used two techniques to normalize gene measurements. First, we used the average of the cycles from the same three housekeeping genes as the control within the array. We then subtracted the number of cycles of a target gene from the average of the housekeeping genes. Reported Ct values of 34 or greater were considered to be unidentified or unexpressed genes. This method is demonstrated in Fig. 1 was used to correlate gene expression levels for each of the 91 genes measured by the two different technologies -- Affymetrix and TaqMan.

TaqMan qPCR-based expression levels passed through a quality control step and were normalized using housekeeping genes. Reported Ct values of 34 or greater were considered to be unexpressed and therefore were not considered in the analysis of a given pathway on a subject-by-subject basis. The average of the housekeeping genes GAPDH, HPRT1 and GUSB served as an endogenous control for the assay. Noting that large Ct values imply less gene sample expression, we subtracted each probe’s ΔCt value from the control to obtain a Ct score that rises with expression consistent with the array-based expression measurement.

### Calibration and PSRP 91 gene computation

Each TaqMan qPCR assay is normalized so it requires no control outside of its own assay. However there are technology-specific differences between the Affymetrix and TaqMan probes. We accounted for this in a calibration step: In deriving the array-based measurements we relied on mean zero, standard deviation one (scaled and centered) values for constructing the risk indexes that comprise the Patient-Specific Risk Profile (PSRP)^1^. We also normalized the TaqMan qPCR expression values by scaling and centering using the observed probe average and standard deviation (Additional file 1: Table S3). Thus a new sample can be normalized and calibrated and the PSRP 91 gene indexes can be computed.

### Statistical methods

For each sample, we used Pearson and Spearman’s rank correlation to compare measurements. The curves for each patient’s 91 gene expression measurements are provided in Fig. 1. We used a locally weighted scatterplot smoothing (LOWESS) to obtain a summary of the relationship between gene expression derived by one technology and that derived by the other.

## Results

### RNA quality metrics and dropped genes

The quality of the RNA derived from the snap-frozen tissues was uniformly high with a RNA quality indicator (RQI) of >7 for 20 of the 28 specimens. The remaining 8 specimens were all 6.2 or greater, except a single outlier with a RQI of 2.9. The FFPE samples had predictably low RQI levels ranging from 1.9 to 3.1. The range of storage time of the tissue samples did not correla te with RQI for the FFPE samples. TaqMan probes for *WNT16*, *WNT3*, and *DNTT* had ΔCt of >34 and were considered unexpressed in most assays (84 % unexpressed, 94 % unexpressed, and 100 % unexpressed respectively); *PLA2G2D* and *CACNG1* were not expressed in 37 % and 46 % of assays respectively, and 15 other probes were not expressed at least once in 68 total TaqMan assays (Additional file 1: Table S4). These findings are consistent with the data showing that these were among the lowest expressing genes in TCGA samples. Samples with low RQI levels still exhibited reportable gene expression levels. Probes considered unexpressed (Ct >34) were removed from samples on a per-assay basis and were not included in the denominator for the index calculation.

### Gene-to-gene comparison of expression levels measured by Affymetrix microarrays and TaqMan qPCR

From TCGA, we obtained gene expression levels in snap-frozen ovarian cancer samples from patients treated at Washington University (St. Louis, MO). To evaluate the concordance of expression measured by array-based probes and qPCR-based probes we acquired ovarian carcinoma samples extracted from the same patient and case, using TCGA numbers for identification. When the microarray and qPCR outputs were plotted against each other and matched gene for gene across the patients, the overall correlation was r = 0.60 (Fig. 1). The plotted slope confirms that the two techniques gave equivalent expression levels and that higher expression of a target gene resulted in a higher number (arbitrary value) on the y-axis. Taking the 10 specimens individually and performing a per-patient smooth estimate of the output (Fig. 1) showed a consistent correlation across the various genes measured. Using housekeeping genes to normalize expression of both technologies also demonstrated that the expression levels of the 91 genes of interest were similar across the two measurement platforms.

### Validating TaqMan assay profiles between matched paraffin-embedded and snap frozen samples

**Fig. 2:**
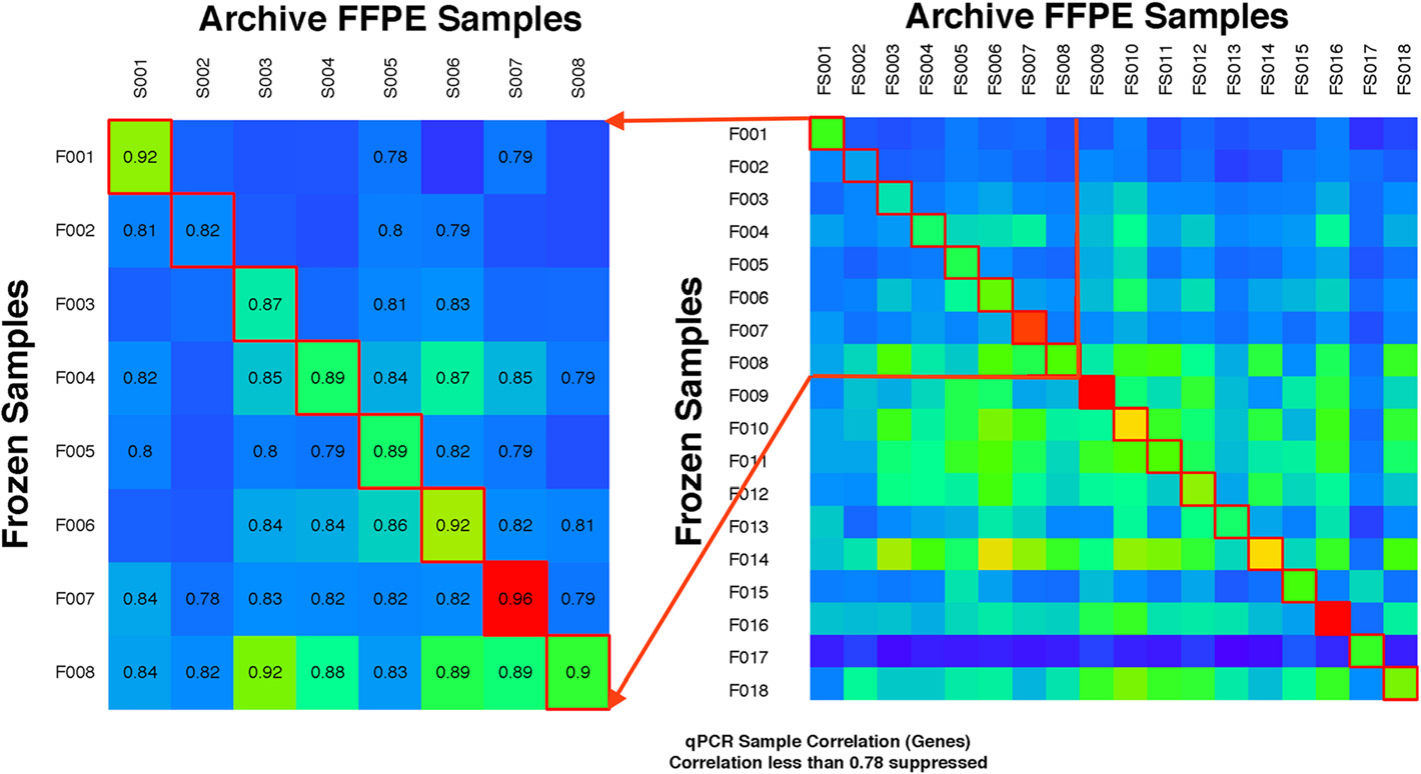
Correlation of gene expression from 18 matched serous ovarian cancer samples. F-labeled samples represent snap-frozen tissue; S represents each patient’s matched FFPE tissue block. All samples were obtained from an initial surgical procedure, and gene outputs were measured with qPCR. Blue represents lower correlation, red higher. An expansion of samples 001 through 008 with absolute level of correlation is provided. Levels of correlation <0.79 are not displayed

To gauge whether our PSRP 91 gene TaqMan qPCR assay provides equivalent expression measurements from both snap-frozen and paraffin-embedded samples, we measured gene expression in the tumor-matched samples of the 18 patients at the Medical College of Wisconsin who had tumor tissue preserved by both snap- freezing and the standard FFPE. The expression outputs were correlated for each gene between the two preservation techniques for each patient. Intra-patient gene expression was higher than any inter-patient gene expression (Fig. 2). Eight of the patients are highlighted in Fig. 2, and the degree of correlation across genes is noted. The correlation between preservation techniques was excellent: > = 0.82 per pair of matched samples. The range of correlation among the 18 sample sets was from 0.82 to 0.96 when comparing snap frozen to FFPE samples. A qPCR was repeated on a second cut from the snap frozen specimens, and the correlation ranged from 0.84 to 0.97 for the 18 samples demonstrating a high level of reproducibility.

### Distribution of pathway expression from 18 FFPE samples measured with qPCR and mapped to the TCGA cohort

**Fig. 3:**
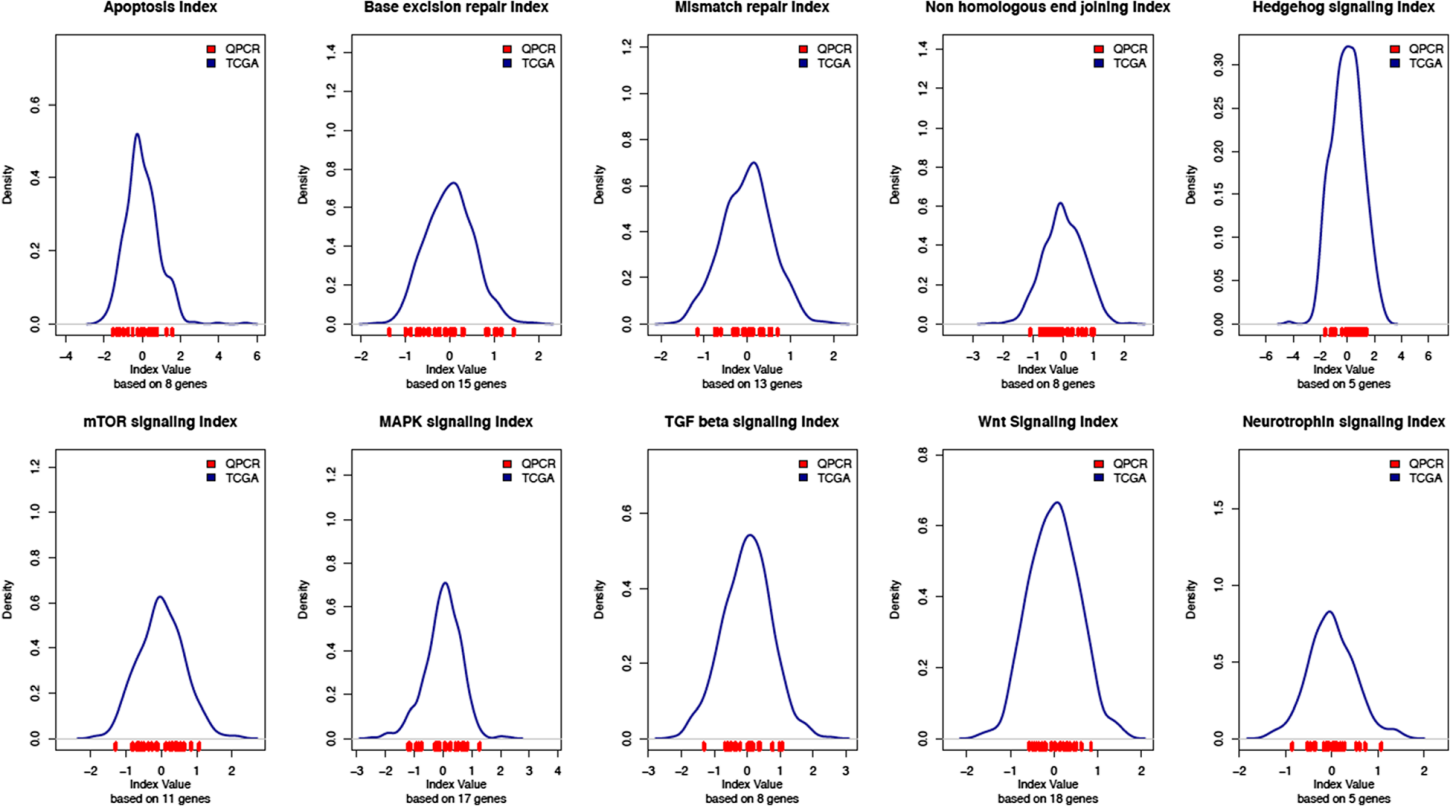
Range of gene expression measured from the selected pathways. The bell curve represents the distribution of expression across the entire TCGA cohort, using Affymetrix array. The red ticks on the x-axis represent gene expression levels aggregated within a pathway from the 18 patients whose FFPE samples were measured using qPCR. The range of expression can be normalized across the Affymetrix and TaqMan qPCR platforms. Patient samples measured with qPCR have a range of expression that does not appear biased within the normal curve

Plotting the distribution of Affymetrix pathway expression for patients from the TCGA gave a normal curve. Samples measured by qPCR (noted with red ticks in Fig. 3) centered under these normal curves. Figure 3 shows the TCGA-generated expression distribution for each pathway. The distribution of each of the 18 samples we measured by qPCR falls within the normal curves, and does not appear biased when we observe their position under the Affymetrix-generated curves for each of the 10 pathways. The PSRP 91 gene assay utilizes gene expression aggregated within a pathway to stratify outcomes. Distribution of these aggregations is not biased by changes in technology or preservation.

## Discussion

This manuscript demonstrates the feasibility of comparing measurements of gene expression used to model clinical outcomes in ovarian cancers from alternative technology and tissue preservation. We demonstrated that qPCR outputs of high-grade serous ovarian carcinoma specimens were nearly identical whether tissue was preserved by snap freezing or fixed with formalin and embedded in a paraffin block. This work was driven by the discovery of a gene panel that is predictive of ovarian cancer patient survival and response to therapy. The correlations described here will enable us to better test our modeling in archived FFPE tissue samples.

A similar correlation between fresh-freezing and FFPE has been observed with breast cancer samples, but a cDNA-mediated annealing, selection, extension, and ligation (DASL) platform was used for gene measurement [10]. That correlation was observed across an unselected whole genome assay when the data was median centered, a technique similar to ours. Interestingly, these authors noted a high level of concordance between tissue types when they applied a selected predictive model that used 291 genes. When ovarian tissue gene expression in 240 FFPE samples was measured using DASL, the correlation was 0.618 to gene signatures described by Tothill and a TCGA working group [9], a high enough level to allow for preservation of the predictive value of the gene sets. Our predictive technique relies less on direct gene-to-gene comparison, as a group of genes is evaluated within a pathway. However, we observed a similar gene-to-gene correlation for our selected set (r = 0.60). A second qPCR run of the snap frozen samples against the FFPE demonstrated that the measurements were stable.

Large variations in expression have been noted to provide confidence in cross-technological measurements. Fedorowicz found correlation between fresh- frozen and FFPE ovarian cancer samples, but that study was confined to the top 100 differentially expressed genes [7]. In contrast, our PSRP 91 gene TaqMan assay did not require genes to have high levels of differential. Although we observed lower expression genes drop out occasionally (e.g., 16 % of the WNT16 assays), this did not seem to offer significant changes at the level of pathway expression. The degree this drop out effects the overall prediction capacity of the model needs further clarification.

We used a combination of two techniques to normalize gene expression across the two technological platforms. First, we averaged housekeeping genes and simply subtracted the result from the target gene in both technologies. Second, we used the scale and center technique that is commonly used in Affymetrix analyses. To ensure the expression levels of the TaqMan data were on the same scale as those from the TCGA Affymetrix data, TaqMan qPCR outputs were centered and scaled. After that normalization they showed comparable ranges of expression.

Variations in expression levels reported from this study are within previously described tolerances [11–14], and the range of differences over an entire pathways appears to have a potentially small or negligible effect on predictive power. Thus, our method for applying the Patient-Specific Risk Profile that was derived with snap-frozen tissue and large-scale Affymetrix microarrays can be effectively applied to a limited gene set measured by qPCR, using RNA extracted from FFPE tissues. Our PSRP 91 gene assay uses measurements aggregated within a cellular pathway, with unbiased selection techniques. This allows poorly expressed genes to be weighted as much as highly expressed genes in the predictive model.

Our work was limited by the small sample size of the snap-frozen tissues we obtained to compare to outputs provided by TCGA. Moreover, we did not measure gene expression in samples preserved for more than 3 years when we compared the FFPE blocks to the snap-frozen samples. The inability to detect expression changes as FFPE blocks age has been a concern in prior reports, but improved techniques and choices in housekeeping genes appear to have reduced its potential impact [15]. Variations in pathologic processing and the ischemia in the tissue sample may be a source of noise in the FFPE gene measurements. Thus far, our assessment of RQI showed lower quality for these specimens, which is expected. This did not seem to effect the quality of measurement for a plurality of the genes assayed. Fixation for snap frozen and FFPE samples occurred simultaneously after surgical removal. Another concern is intra-tumor heterogeneity in duplicate patient samples. Variation in a single patient’s tumor profiling has been identified by groups measuring expression arrays from multiple tumor sites. Our comparisons were from the same tumor excised at the same time, but variation in location may affect the correlation of the two tumor sites [16]. The loss of detection of some lower expressing genes in the qPCR (e.g., WNT16) is a concern for future modeling. Alternative technologies may need to be considered depending on the weighted importance of specific genes.

In summary, this study validated the use of FFPE tissue and qPCR—instead of snap-frozen tissue and microarrays—to obtain gene expression data for core cellular pathways. This supports the use these tissue samples when predictive modeling of ovarian cancer was done in larger data sets such as the TCGA. The variation in expression noted between our different samples does not appear to significantly distort expected outputs, leading us to believe that a model derived from expression reported using one approach could be used with a more convenient and “real world” approach when evaluating clinical samples. Our assay will be tested in a recurrent disease setting to more definitively evaluate predictive capacity prospectively.

## Conclusion

This study offers evidence that a predictor model based on a large data set generated from Affymetrix microarray and snap frozen ovarian carcinoma samples can be applied to paraffin embedded clinical samples from a local pathology lab. Our predictive model used a “pathway” approach, and we observed that these local samples had pathway measurements across 10 separate pathways that fell within expected ranges. Normalization with scaling and centering was used for this in the qPCR data generated.

## Additional file

Additional file 1: Table S1. Gene list. **Table S2.** Pathway list and genes in the pathway. **Table S3.** Scale and center data used for qPCR. **Table S4.** Genes unexpressed in at least one TaqMan assay. (DOCX 54 kb)

## Abbreviations

TCGA: The Cancer Genome Atlas
qPCR: Quantitative real-time reverse transcription polymerase chain reaction
FFPE: Formalin fixed, paraffin embedded
PSRP: Patient specific risk profile
MAQC: Microarray quality control
GAPDH: Glyceraldehyde 3-phosphate dehydrogenase
HPRT1: Hypoxanthine phosphoribosyltransferase 1
GUSB: beta-D-glucuronidase
LOWESS: Locally weighted scatterplot smoothing
DASL: cDNA-mediated Annealing, Selection, extension, and Ligation
