# Supplementary material for: Comparing gene expression data from formalin-fixed, paraffin embedded tissues and qPCR with that from snap-frozen tissue and microarrays for modeling outcomes of patients with ovarian carcinoma
Source: BMC Clin Pathol. 2015 Sep 24;15:17. doi: 10.1186/s12907-015-0017-1 (PMC4582729; doi:10.1186/s12907-015-0017-1)
Supplement: Additional file 1: Table S1. — Gene list. Table S2. Pathway list and genes in the pathway. Table S3. Scale and center data used for qPCR. Table S4. Genes unexpressed in at least one TaqMan assay. (DOCX 54 kb) [file 12907_2015_17_MOESM1_ESM.docx]

Additional file 1

Table S1

Gene list

| Symbol | affyname | unigene | entrezid |
| --- | --- | --- | --- |
| ACVR2A | 205327_s_at | Hs.470174 | 92 |
| AIFM1 | 205512_s_at | Hs.424932 | 9131 |
| AKT1 | 207163_s_at | Hs.525622 | 207 |
| AKT2 | 203808_at | Hs.631535 | 208 |
| AKT3 | 212607_at | Hs.498292 | 10000 |
| APAF1 | 204859_s_at | Hs.728891 | 317 |
| APC | 203525_s_at | Hs.158932 | 324 |
| APEX1 | 210027_s_at | Hs.73722 | 328 |
| BID | 204493_at | Hs.591054 | 637 |
| BMPR2 | 209920_at | Hs.471119 | 659 |
| CACNA1D | 207998_s_at | Hs.476358 | 776 |
| CACNB2 | 207776_s_at | Hs.59093 | 783 |
| CACNG1 | 206612_at | Hs.147989 | 786 |
| CALM3 | 200622_x_at | Hs.515487,Hs.706125,Hs.722012 | 808 |
| CAMK2G | 212669_at | Hs.523045 | 818 |
| CASP8 | 207686_s_at | Hs.599762 | 841 |
| CDC25B | 201853_s_at | Hs.153752 | 994 |
| CSNK1E | 202332_at | Hs.474833 | 1454 |
| CSNK1G3 | 220768_s_at | Hs.129206 | 1456 |
| CSNK2A1 | 206075_s_at | Hs.644056 | 1457 |
| DDIT4 | 202887_s_at | Hs.523012 | 54541 |
| DNTT | 210487_at | Hs.534206 | 1791 |
| DUSP1 | 201041_s_at | Hs.171695 | 1843 |
| DUSP8 | 206374_at | Hs.41688 | 1850 |
| FEN1 | 204767_s_at | Hs.409065 | 2237 |
| FZD10 | 219764_at | Hs.31664 | 11211 |
| FZD3 | 219683_at | Hs.40735 | 7976 |
| GAS1 | 204456_s_at | Hs.65029 | 2619 |
| HSPA1L | 210189_at | Hs.690634 | 3305 |
| ID1 | 208937_s_at | Hs.504609 | 3397 |
| ID4 | 209291_at | Hs.519601 | 3400 |
| JUN | 201464_x_at | Hs.696684 | 3725 |
| KRAS | 204009_s_at | Hs.505033 | 3845 |
| LEF1 | 210948_s_at | Hs.726506 | 51176 |
| LIG3 | 204123_at | Hs.100299 | 3980 |
| LIG4 | 206235_at | Hs.166091 | 3981 |
| LTBP1 | 202728_s_at | Hs.619315 | 4052 |
| MAP3K4 | 204089_x_at | Hs.390428 | 4216 |
| MAPT | 203928_x_at | Hs.101174 | 4137 |
| MBD4 | 209579_s_at | Hs.35947 | 8930 |
| MLH3 | 204838_s_at | Hs.436650 | 27030 |
| MLST8 | 220587_s_at | Hs.29203 | 64223 |
| MRE11A | 205395_s_at | Hs.192649 | 4361 |
| MSH2 | 209421_at | Hs.597656 | 4436 |
| MSH6 | 202911_at | Hs.445052 | 2956 |
| MYC | 202431_s_at | Hs.202453 | 4609 |
| MYD88 | 209124_at | Hs.82116 | 4615 |
| NHEJ1 | 219418_at | Hs.225988 | 79840 |
| NTHL1 | 209731_at | Hs.66196 | 4913 |
| OGG1 | 205301_s_at | Hs.380271 | 4968 |
| PAK1 | 209615_s_at | Hs.435714 | 5058 |
| PARP3 | 209940_at | Hs.271742 | 10039 |
| PARP4 | 202239_at | Hs.117825 | 143 |
| PDGFA | 205463_s_at | Hs.535898 | 5154 |
| PIK3R5 | 220566_at | Hs.278901 | 23533 |
| PLA2G2D | 220423_at | Hs.189507 | 26279 |
| POLB | 203616_at | Hs.654484 | 5423 |
| POLD1 | 203422_at | Hs.279413 | 5424 |
| POLD2 | 201115_at | Hs.306791 | 5425 |
| POLD3 | 212836_at | Hs.82502 | 10714 |
| POLD4 | 202996_at | Hs.523829 | 57804 |
| POLL | 221049_s_at | Hs.523230 | 27343 |
| PORCN | 219483_s_at | Hs.386453 | 64840 |
| PPP3CA | 202425_x_at | Hs.435512 | 5530 |
| PRKACA | 202801_at | Hs.631630 | 5566 |
| PRKCA | 206923_at | Hs.531704,Hs.708867 | 5578 |
| RAC1 | 208640_at | Hs.413812 | 5879 |
| RFC2 | 1053_at | Hs.647062 | 5982 |
| RFC3 | 204127_at | Hs.115474 | 5983 |
| RFC4 | 204023_at | Hs.714318 | 5984 |
| RFC5 | 203209_at | Hs.506985 | 5985 |
| ROCK1 | 213044_at | Hs.306307 | 6093 |
| RPA3 | 209507_at | Hs.487540 | 6119 |
| RPS6KA2 | 204906_at | Hs.655277 | 6196 |
| RPS6KA3 | 203843_at | Hs.445387 | 6197 |
| RPS6KB2 | 203777_s_at | Hs.534345 | 6199 |
| SMUG1 | 218685_s_at | Hs.632721 | 23583 |
| SSBP1 | 202591_s_at | Hs.490394 | 6742 |
| STK11 | 204292_x_at | Hs.515005 | 6794 |
| STK3 | 204068_at | Hs.492333 | 6788 |
| TDG | 203742_s_at | Hs.584809 | 6996 |
| TNFRSF10D | 210654_at | Hs.213467 | 8793 |
| ULK1 | 209333_at | Hs.47061 | 8408 |
| UNG | 202330_s_at | Hs.191334 | 7374 |
| WNT16 | 221113_s_at | Hs.272375 | 51384 |
| WNT3 | 221455_s_at | Hs.445884 | 7473 |
| WNT5A | 205990_s_at | Hs.643085 | 7474 |
| WNT5B | 221029_s_at | Hs.306051 | 81029 |
| XRCC4 | 205071_x_at | Hs.567359 | 7518 |
| XRCC5 | 208642_s_at | Hs.388739 | 7520 |
| YWHAB | 208743_s_at | Hs.643544 | 7529 |

Table S2

Pathway list and genes in the pathway

| Pathway Name | Genes measured in each Pathway |
| --- | --- |
| Apoptosis | AIFM1, AKT2, APAF1, CASP8, MYD88, PPP3CA, TNFRSF10D |
| Base Excision Repair | APEX1, FEN1, LIG3, MBD4, MBD4, MTHL1, NTHL1, OGG1, PARP3, PARP4, PARP5, POLB, POLD1, POLD2, POLD3, SMUG1, TDG |
| Mismatch Repair | MLH 3, MSH2, POLD1, POLD2, POLD3, POLD4, RFC2, RFC3, RFC4, RFC5, RPA3, SSBP1, |
| Non-homologous End Joining | DNTT, FEN1, LIG4, MRE11A, NHEJ1, POLL, XRCC4, XRCC5 |
| Hedgehog | CSNK1E, CSNK1G3, GAS1, WNT5A, WNT5B |
| mTOR | AKT1, AKT2, AKT3, DDIT4, PIK3R5, PS6KA2, RPS6KA3, STK11, ULK1 |
| MAPK | AKT2, AKT3, CACNA1D , CACNB2, CDC25B, CACNG1, DUSP1, DUSP8, MAP3K4, MAPT, MYC, PDGFA, PAK1, PLA2G2D, PPP3CA , PRKACA , RPS6KA, STK3 |
| TGF Beta | ACVR2A, BMPR2, ID1, ID4, LTBP1, MYC, PPP3CA , PRKACA , RAC1, ROCK1, WNT3, WNT5B, WNT16 |
| Wnt | APC, CAMK2G, CSNK2A1, CSNK1E, FZD3, FZD10, JUN, LEF1, MYC, PORCN, PPP3CA, PRKACA, PRKCA, RAC1, ROCK1, WNT3 |
| Neurotrophin | AKT2, CALM3, KRAS, RAC1, RPS6KA2, YWHAB |

Table S3

Scale and center data used for qPCR

|  | Center | Std.Error | Scale | Std.Error |
| --- | --- | --- | --- | --- |
| GAPDH | 2.940976666 | 0.178824453 | 0.799727267 | 0.461722753 |
| HPRT1 | -1.882596465 | 0.170044393 | 0.760461644 | 0.439052735 |
| GUSB | -1.058380201 | 0.12796913 | 0.572295349 | 0.330414874 |
| ACVR2A | -3.194362705 | 0.191580518 | 0.856774121 | 0.494658769 |
| AIFM1 | -1.305356513 | 0.235836837 | 1.054694399 | 0.608928095 |
| AKT1 | -1.859865567 | 0.47710825 | 2.133692958 | 1.231888203 |
| AKT2 | -3.701283427 | 0.590533053 | 2.640944099 | 1.524749787 |
| AKT3 | -4.98122216 | 0.428006984 | 1.914105423 | 1.105109281 |
| APAF1 | -4.814581642 | 0.328139244 | 1.467483309 | 0.847251884 |
| APC | -5.331101822 | 0.136170511 | 0.608973038 | 0.351590747 |
| APEX1 | -0.932754592 | 0.353930343 | 1.582824611 | 0.913844215 |
| BID | -3.357529959 | 0.122722083 | 0.548829839 | 0.316867055 |
| BMPR2 | -2.51351824A9 | 0.29236768 | 1.307508013 | 0.754890103 |
| CACNA1D | -7.099287179 | 0.343357789 | 1.456743728 | 0.866185003 |
| CACNB2 | -7.3321471 | 0.383916034 | 1.628817785 | 0.968500849 |
| CACNG1 | -7.386615903 | 0.55978589 | 2.239143561 | 1.376598934 |
| CALM3 | -1.766214383 | 0.336158405 | 1.503346092 | 0.867957271 |
| CAMK2G | -2.923875178 | 0.422555869 | 1.889727296 | 1.091034563 |
| CASP8 | -3.237886251 | 0.207452235 | 0.927754598 | 0.535639367 |
| CDC25B | -3.572280945 | 0.207855166 | 0.929556561 | 0.536679731 |
| CSNK1E | -1.563838787 | 0.421660353 | 1.885722426 | 1.08872235 |
| CSNK1G3 | -2.982523011 | 0.218776366 | 0.978397654 | 0.564878149 |
| CSNK2A1 | -1.766412895 | 0.288764014 | 1.29139193 | 0.745585478 |
| DDIT4 | -1.851437339 | 0.286138014 | 1.279648101 | 0.738805176 |
| DNTT | -6.375060176 | 0.097886631 | 0.239772298 | 0.201623666 |
| DUSP1 | -2.066678673 | 0.452618575 | 2.024171804 | 1.168656136 |
| DUSP8 | -5.847200985 | 0.240799135 | 1.076886471 | 0.621740694 |
| FEN1 | -4.192265123 | 0.230074139 | 1.028922829 | 0.594048872 |
| FZD10 | -4.895503132 | 0.527827523 | 2.239382526 | 1.331544817 |
| FZD3 | -3.632929445 | 0.297009616 | 1.328267384 | 0.766875532 |
| GAS1 | -2.757203874 | 0.424658081 | 1.899128672 | 1.09646245 |
| HSPA1L | -4.966507699 | 0.224659065 | 1.004705882 | 0.580067211 |
| ID1 | -3.613050441 | 0.366873662 | 1.640708897 | 0.947263723 |
| ID4 | -1.838369741 | 0.276034527 | 1.234463931 | 0.712718083 |
| JUN | -3.11168036 | 0.261940364 | 1.171432922 | 0.676327113 |
| KRAS | -2.848445674 | 0.278157992 | 1.243960358 | 0.718200847 |
| LEF1 | -3.977946539 | 0.438853311 | 1.962611669 | 1.133114375 |
| LIG3 | -3.784287314 | 0.382397083 | 1.710131743 | 0.987345022 |
| LIG4 | -5.463697256 | 0.184518127 | 0.825190151 | 0.476423756 |
| LTBP1 | -2.43248651 | 0.329950897 | 1.475585268 | 0.851929552 |
| MAP3K4 | -4.12836383 | 0.189958342 | 0.849519531 | 0.49047033 |
| MAPT | -6.796206197 | 0.264220437 | 1.181629719 | 0.682214236 |
| MBD4 | -2.031715103 | 0.136158861 | 0.608920937 | 0.351560667 |
| MLH3 | -5.125114735 | 0.138005677 | 0.617180152 | 0.356329127 |
| GBL | -2.80584081 | 0.344765302 | 1.541837302 | 0.890180181 |
| MRE11A | -3.466865554 | 0.376074766 | 1.681857483 | 0.971020871 |
| MSH2 | -1.861558326 | 0.268272915 | 1.19975295 | 0.692677689 |
| MSH6 | -2.676686091 | 0.300748919 | 1.344990054 | 0.776530369 |
| MYC | -0.455083589 | 0.335345451 | 1.49971045 | 0.865858232 |
| MYD88 | -1.853269355 | 0.148792642 | 0.665420923 | 0.384180949 |
| NHEJ1 | -4.043915174 | 0.183154265 | 0.819090774 | 0.472902279 |
| NTHL1 | -3.961791792 | 0.185720397 | 0.830566864 | 0.479528002 |
| OGG1 | -3.027842894 | 0.41499709 | 1.855923405 | 1.071517878 |
| PAK1 | -4.31455037 | 0.31480192 | 1.407836987 | 0.812815064 |
| PARP3 | -4.421795342 | 0.203859533 | 0.911687545 | 0.52636305 |
| PARP4 | -2.35061363 | 0.24660881 | 1.102868127 | 0.63674121 |
| PDGFA | -6.289975415 | 0.435790735 | 1.948915417 | 1.125206841 |
| PIK3R5 | -5.703596558 | 0.147802731 | 0.660993906 | 0.381625009 |
| PLA2G2D | -8.244138116 | 0.649591612 | 2.054189043 | 1.452531002 |
| POLB | -2.143500355 | 0.159223318 | 0.712068327 | 0.41111284 |
| POLD1 | -4.327879803 | 0.151544496 | 0.677727592 | 0.391286207 |
| POLD2 | -2.013191647 | 0.213206262 | 0.953487391 | 0.550496202 |
| POLD3 | -2.868458459 | 0.141479048 | 0.632713539 | 0.365297332 |
| POLD4 | -4.261565475 | 0.161138531 | 0.720633418 | 0.416057898 |
| POLL | -5.596526871 | 0.212641816 | 0.95096311 | 0.549038808 |
| PORCN | -5.320117825 | 0.182051257 | 0.814157972 | 0.470054324 |
| PPP3CA | -2.66438216 | 0.172640006 | 0.772069576 | 0.445754577 |
| PRKACA | -3.120648475 | 0.428099203 | 1.914517839 | 1.10534739 |
| PRKCA | -4.045714317 | 0.400517473 | 1.791168591 | 1.034131668 |
| RAC1 | 0.197202904 | 0.241942747 | 1.082000858 | 0.624693486 |
| RFC2 | -3.658109134 | 0.173400512 | 0.775470663 | 0.447718196 |
| RFC3 | -3.351258005 | 0.229986276 | 1.028529895 | 0.593822012 |
| RFC4 | -1.348477789 | 0.146161261 | 0.653653032 | 0.377386754 |
| RFC5 | -4.549447508 | 0.180002885 | 0.804997375 | 0.464765451 |
| ROCK1 | -2.213319968 | 0.207152276 | 0.926413143 | 0.534864878 |
| RPA3 | -3.665457273 | 0.409819421 | 1.83276817 | 1.058149196 |
| RPS6KA2 | -3.315830991 | 0.344762882 | 1.541826482 | 0.890173935 |
| RPS6KA3 | -2.816465495 | 0.308817997 | 1.381076069 | 0.79736464 |
| RPS6KB2 | -3.712835832 | 0.310048162 | 1.386577531 | 0.800540911 |
| SMUG1 | -4.907448055 | 0.153901272 | 0.688267411 | 0.397371375 |
| SSBP1 | -1.102408025 | 0.277213342 | 1.239735752 | 0.71576177 |
| STK11 | -3.786776446 | 0.375048342 | 1.677267177 | 0.968370656 |
| STK3 | -3.307553302 | 0.311522581 | 1.393171337 | 0.804347846 |
| TDG | -2.603254667 | 0.144832829 | 0.647712103 | 0.373956757 |
| TNFRSF10D | -6.873951582 | 0.229006341 | 1.02414749 | 0.591291829 |
| ULK1 | -3.789800795 | 0.339148477 | 1.516718099 | 0.875677603 |
| UNG | -2.718693546 | 0.248050934 | 1.109317501 | 0.640464758 |
| WNT16 | -6.183792127 | 0.809812831 | 1.983628224 | 1.668025863 |
| WNT3 | -7.06729075 | 0.834394826 | 2.207601206 | 1.75564062 |
| WNT5A | -4.199623046 | 0.288153819 | 1.288663054 | 0.744009961 |
| WNT5B | -5.326462533 | 0.381181789 | 1.617217367 | 0.9616032 |
| XRCC4 | -5.035500702 | 0.224320341 | 1.003191062 | 0.57919263 |
| XRCC5 | 0.237349096 | 0.137760716 | 0.61608465 | 0.355696639 |
| YWHAB | 0.539388932 | 0.135844718 | 0.607516047 | 0.350749553 |

TABLE S4

Genes unexpressed in at least one TaqMan assay

| AKT2 |
| --- |
| CACNB2 |
| CACNG1 |
| DNTT |
| LIG4 |
| MAPT |
| PLA2G2D |
| PIK3R5 |
| PORCN |
| TNRFRSF10D |
| WNT3 |
| WNT5B |
| WNT16 |
